# Prussian Blue Sensor for Bacteria Detection in Personal Protection Clothing

**DOI:** 10.3390/polym15040872

**Published:** 2023-02-10

**Authors:** Liliana Leite, Vânia Pais, João Bessa, Fernando Cunha, Cátia Relvas, Noel Ferreira, Raul Fangueiro

**Affiliations:** 1 Fibrenamics—Institute of Innovation on Fiber-Based Materials and Composites, University of Minho, 4800-058 Guimarães, Portugal; 2 Centre for Textile Science and Technology (2C2T), University of Minho, 4800-058 Guimarães, Portugal; 3 A. Ferreira & Filhos, Rua Amaro de Sousa 408, 4815-901 Caldas de Vizela, Portugal; 4 Department of Textile Engineering, University of Minho, 4800-058 Guimarães, Portugal

**Keywords:** defence materials, personal protective equipment, bacterial sensor, Prussian blue

## Abstract

Biological hazards can be defined as substances that endanger the life of any living organism, most notably humans, and are often referred to as biohazards. Along with the use of personal protective equipment (PPE), early detection of contact is essential for the correct management and resolution of a biological threat, as well as lower mortality rates of those exposed. Herein, Prussian blue (PB) was evaluated as a functional compound applied on polyester knits to act as an on-site sensor for bacteria detection. In order to study the best compound concentration for the intended application, polymeric solutions of 0.5, 1 and 2 g/L were developed. The three conditions tested displayed high abrasion resistance (>2000 cycles). The bacterial sensing capacity of the coated knits was assessed in liquid and solid medium, with the functionalised substrates exhibiting the capability of detecting both Gram-positive and Gram-negative bacteria and changing colours from blue to white. Evaluation of water repellence and chemical penetration resistance and repellence was also performed in polyester functionalised with PB 0.5 and 1 g/L. Both knits showed a hydrophobic behaviour and a capacity to resist to penetration of chemicals and level 3 repellence effect for both acid and base chemicals.

## 1. Introduction

Biological hazards can be defined as substances that endanger the life of any living organism, most notably humans, and are often referred to as biohazards [1]. These agents can be viruses, bacteria and other pathogenic organisms, along with their proteins and toxins [2]. The need to use personal protective equipment (PPE) capable of being the last line of defence against those biological threats is of extreme importance. Thus, the development of protective clothing and textiles as a response to this issue has grown. Protective clothing is essential in many sectors and professions, such as the agricultural and livestock sector, the healthcare sector, laboratory work and in the military and firefighting sector [3,4,5,6]. For example, due to their efficiency and extensive damage, biological agents have been used as weapons since prehistoric times, as warfare agents or in bioterrorism [7]. A few of the most common diseases caused by those agents are anthrax, plague, smallpox and botulism [8]. Furthermore, PPE for hazardous biological agents is also indispensable for laboratory workers and health care personnel whenever there is a risk of exposure. This equipment is important to protect the user against toxic substances derivate from coughs, sneezes, body fluids and experimental work residues. In a hospital scenario, PPE is also crucial to lowering cross-infection risk [9]. During the development of such clothing, it is critical to obtain a protective barrier without sacrificing comfort [3]. For example, the equipment must be hydrophobic, to prevent the contact of contaminated liquids with the skin, and permeable to air and moisture to ensure user welfare and thermo-physiological comfort in long-term use [10].

Along with protection, early detection of contact is essential for the correct management and resolution of a biological threat, as well as lower mortality rates of those exposed [11]. However, despite the efforts for technological development in the area of biosensors throughout the years, most techniques either require the need for specialised apparel and well-trained technicians or cannot be incorporated directly into protective clothing, allowing constant monitorisation for everyday use [2,12,13]. Other approaches for biological sensing, based on the colourimetric and optical response of a compound, have been studied [14,15,16,17]. Prussian blue (PB) (Fe_4_[Fe(CN)_6_]_3_), also known as ferric hexacyanoferrate, is a dark blue pigment applied in various sectors, from the textile industry to its use in biochemical and biomedical areas [17,18,19]. Its capacity to be applied as a compound for bacterial sensing has been reported [20] and is related to a redox mechanism, i.e., the reduction of PB carried out by living bacteria as a consequence of their normal metabolism causes a shift in the colour from blue to white [21]. Accordingly, when applied to textile materials, this alteration causes a change in the colour displayed by the sample. A study completed by Ferrer-Vilanova and collaborators showed the sonochemical coating of polyester–cotton materials with PB-synthesised nanoparticles for further use in hospital textiles [22]. This article demonstrated the textile’s capability to detect bacteria, both Gram-positive and Gram-negative. Jahn et al. (2005) also related the colour changes displayed by PB with the reduction process induced by bacteria [21]. Microbial reduction by *Geobacter metallireducens* and *Shewanella alga* strain BrY lead to the formation of colourless solid minerals and this reaction was reversible after exposure to air.

In this study, commercial PB was used as a functional compound and applied on polyester (PES) knits in an attempt to develop a simple, low cost and direct on-site method to detect biohazards, more specifically bacteria. These products can then be attached to personal protective clothing in the form of patches in strategically selected places, providing a lightweight system. To optimise the formulation, three different concentrations of PB were used and textile application was completed by stamping, already having in mind the process simplification for subsequent industrial production. The samples obtained were then characterised and bacterial sensing assays were performed with Gram-negative (*Escherichia coli*) and Gram-positive bacteria (*Staphylococcus aureus*), both in liquid and solid medium. 

## 2. Materials and Methods

### 2.1. Materials

Prussian blue, pure (Eisen(III)-hexacyanoferrat(II)) was obtained from Thermo Scientific (Waltham, MA, USA). The polyurethane polymeric base, Edolan SN and Thickener A02 were purchased from ADI Center Portugal (Santo Tirso, Portugal). Polyester knits were provided by A. Ferreira & Filhos (Caldas de Vizela, Portugal). The knits tested are composed of 100% polyester, constructed by the flat knitting machine gauge 14 with a thickness of 2.3 mm and a mass per unit area of 944 g/m^2^. Sodium borohydride (NaBH_4_) was obtained from TCI. 2-Morpholinoethanesulfonic acid monohydrate (MES) was purchased from Merck (Darmstadt, Germany). Tryptic soy broth was acquired from Liofilchem (Roseto degli Abruzzi (TE), Italy). Glucose was purchased from Sigma-Aldrich (St. Louis, MO, USA). Sodium chloride (NaCl), potassium chloride (KCl), sodium phosphate dibasic (Na_2_HPO_4_) and monopotassium phosphate (KH_2_PO_4_) used to prepare a stock solution of 10× PBS buffer were obtained from Sigma.

### 2.2. Preparation of Prussian Blue Formulations and Application on Polyester Knits

Commercial PB was dissolved in Edolan SN and placed under vigorous magnetic agitation for 1 h. Before applying to textiles, 1% (*w*/*v*) of Thickener A02 was added to the polymeric solution and agitated by mechanical stirring for 10 min. The formulation was then stamped on polyester (PES) using a stamping table Zimmer Magnet System Plus and two layers of the polymeric solution were used for each sample. This method is called bar-coating and consists of spreading an excess of solution across the substrate with a long cylindrical bar. Lastly, textile samples were dried at 100 °C for 5 min. In this study, different concentrations of PB were evaluated (0.5 g/L, 1 g/L and 2 g/L). 

### 2.3. Morphological Characterisation of Prussian Blue Functionalised Knits

To analyse the deposition of the coating on the fibrous substrates, polyester fibres were observed using the equipment Leica DM750 M optical microscope with a high-definition camera. For all samples, at least two different zones were observed at a total magnification of 50.

### 2.4. Characterisation of Prussian Blue Functionalised Knits for Air-Permeability and Abrasion Resistance

To perform an indirect evaluation of the material porosity and breathability, the airflow capable of passing through the textile samples was measured using an adaptation of the ISO 9237 standard. This parameter was determined with a TEXTEST Instruments air permeability tester III FX 3300 with a pressure of 200 Pa and a head area of 20 cm^2^. To compare the functionalised samples, polyester control samples were also evaluated.

The assessment of the abrasion resistance was performed according to the standard EN 530 and consisted of using a circular textile sample from the area where the coating was applied with a diameter of 127 mm. Wool abradant fabric from SDC Enterprises Limited was used as an abradant. The Martindale equipment was employed to simulate the constant abrasion that the clothes suffer throughout their use. For this assay, 3000 cycles were completed, following the standard EN ISO 14325, which refers that level 6 protective clothing must be resistant to at least 2000 abrasion cycles. A cycle can be defined as one full rotation with a pressure of 9 kPa and a rotational frequency of 44.5 ± 2.4 min^−1^. Weight loss percentage was calculated based on the initial and final weight of the samples. 

### 2.5. Preliminary Assessment of Prussian Blue Coated Textiles Response to a Reducing Agent

Since the mechanism for bacteria detection is based on a reduction of PB, a preliminary test was performed to verify the activity of the functionalised textiles. Sodium borohydride (1 M NaBH_4_) was used as a reducing agent and PES samples functionalised with 1 g/L of PB were submerged in the solution for 20 h. As a control, the response of the knits to distilled water was also verified. ImageJ software was used to determine the RGB (red, green and blue) values from the images. The RGB model is a combination of red, green and blue to generate a diversity of colours. Each of the primary colours corresponds to a value between 0 and 255. The colour black is obtained when all coordinates correspond to 0, and the white colour is achieved if they correspond to 255 [23]. Image acquisition was performed in environmental conditions with a digital camera Canon PowerShot SX530 HS, with 16 megapixels resolution and 4.3 mm focal length.

### 2.6. Bacterial Culture Preparations

*Escherichia coli* (ATCC 25922) (*E. coli*) and *Staphylococcus aureus* (ATCC 6538) (*S. aureus*) were selected as a model for Gram-negative and Gram-positive bacteria, respectively. The inoculum was prepared by adding a portion of both bacteria in a sterile tryptic soy broth (TSB). Then, the bacteria were incubated aerobically overnight (18 h) at 37 °C with shaking. To adjust bacterial concentration the suspension was centrifugated at 4000 rpm for 10 min (Refrigerated Centrifuge 1248R, Gyrozen), re-suspended in 1× PBS (pH 7.4) and the optical density at 600 nm was measured in a spectrophotometer (EZ Read 400 Microplate Reader). An optical density of 0.1 A.U. was considered equivalent to 10^7^ CFU/mL.

### 2.7. Evaluation of the Bacterial-Sensing Activity of Polyester Textiles Functionalised with Prussian Blue in Liquid and Solid Conditions

Bacterial-sensing activity in liquid conditions was evaluated according to previous research [22]. In this assay, after concentration adjustment in PBS to 10^8^ CFU/mL, the bacterial suspension was centrifugated at 4000 rpm for 10 min and re-suspended in 0.1 M MES buffer (pH 6.1) supplemented with 0.1% (*w*/*v*) glucose. After that, 1 cm^2^ polyester samples functionalised with PB (0.5 g/L, 1 g/L and 2 g/L) were submerged in the bacterial suspension. As controls, one sample for each concentration was put in contact only with MES buffer and one was left in contact with air. For each sample, the test was performed in triplicate. Samples’ colours were analysed after 24 h, 48 h and 120 h and the red channel from the RGB model was measured with ImageJ software. This channel was chosen based on the preliminary tests described above since it was the most sensitive. Image acquisition was performed in environmental conditions, inside the laminar flow hood, with a digital camera Canon PowerShot SX530 HS, with 16 megapixels resolution and 4.3 mm focal length.

To evaluate bacterial-sensing activity in a solid medium, 0.1 mL of the bacterial solution (10^5^ CFU/mL) was spread on a Petri dish containing 15 mL of tryptic soy agar (TSA). After drying, samples of 1 cm^2^ were placed on the agar surface and the Petri dishes were incubated at 37 °C. As a control, a condition using only TSA was introduced. For each sample, the test was performed in triplicate. Assessment of colour change was performed after 24 h of incubation time, using the same method previously explained for liquid conditions.

### 2.8. Evaluation of Water Repellence and Chemical Penetration Resistance and Repellence 

Considering the desired application of the patches on personal protective equipment, tests were performed to evaluate the ability of the materials to repel and resist to the penetration of water and chemical solutions. 

To study water repellence, sample contact angles were evaluated, using a water contact angle test, which measured the angle between a water drop and the sample surface. A volume of 5 µL per water drop was used, with a flow rate of 10 µL/s. A goniometer contact angle system OCA 15 was connected to a digital microscope with a camera to perform this test. The measurements were taken in various parts of the same textile sample. Polyester control knits were also evaluated before functionalisation.

For chemical agents, an assay was performed by adapting the standard ISO 6530, which intends to measure materials’ penetration and repellence indexes against liquid chemicals, especially those with low volatility. Each sample with 5 cm^2^ was arranged on a platform inclined at 45 degrees and placed on top of a filter paper with the same area. After that, 10 mL of liquid was poured into the sample. An acid, H_2_SO_4_ 30% (*v*/*v*), and a base, NaOH 10% (*w*/*v*), were used as chemical agents. According to ISO 14325, penetration and absorption indexes were evaluated and a performance level was attributed. The correlation can be seen in Table 1.

### 2.9. Statistical Analysis

GraphPad Prism 6.01 was used to perform statistical analysis on the results obtained throughout this paper. The results are shown as the average of the replicates performed and the standard deviation. A one-way ANOVA test was used to analyse the results, and Šídák’s test was used to perform multiple comparisons. Throughout the study, a critical value for significance of *p* < 0.05 was used.

## 3. Results and Discussion

### 3.1. Morphological Characterisation of Prussian Blue Functionalised Knits

**Figure 1:**
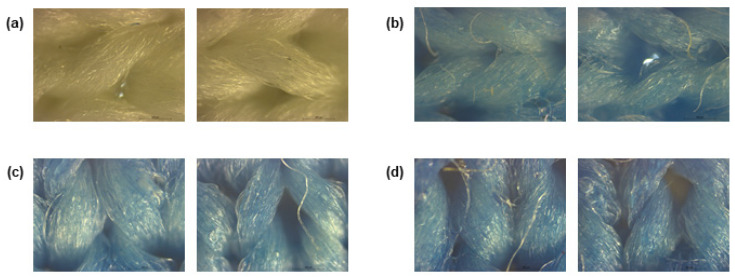
Polyester fibres observed under a microscope at a total magnification of 50, (**a**) before coating and after coating with Prussian blue with a concentration of (**b**) 0.5 g/L, (**c**) 1 g/L and (**d**) 2 g/L.

After stamping, to visualise coating deposition, the fabrics functionalised with PB were subjected to a morphological characterisation by observing them under an optical microscope. As demonstrated in Figure 1, the three concentrations of PB tested generated a uniform coating on the fabrics, even if some naked fibres are observed. As expected, compared to the control, the functional formulations gave a blue colour to the substrate, intensified with increasing concentration.

### 3.2. Characterisation of Prussian Blue Functionalised Knits for Air-Permeability and Abrasion Resistance

The development of personal protective equipment demands a great balance between comfort and protection. Having this in mind, the textiles were characterised for air permeability, since this characteristic is highly related to this equilibrium. Furthermore, abrasion resistance was also characterised due to its importance to ensure the textiles can maintain their properties and aesthetics during their lifetime.

**Figure 2:**
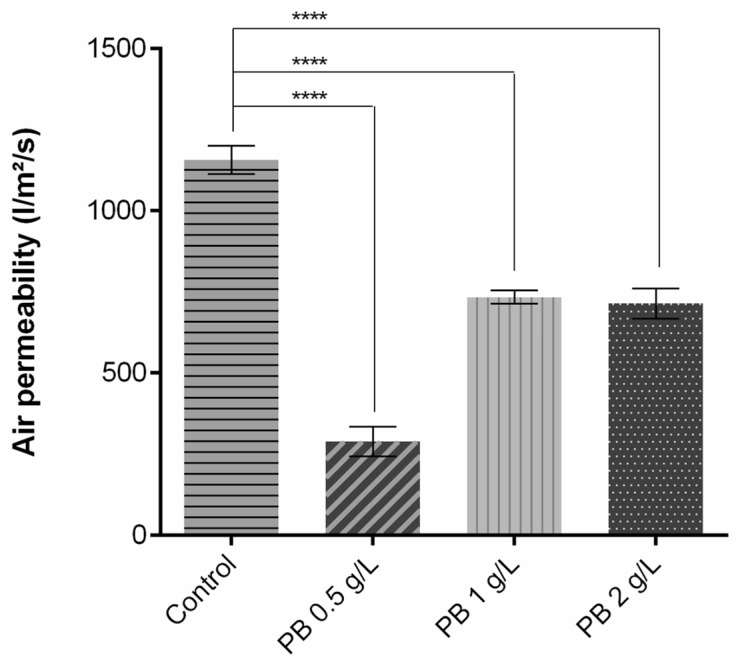
Air permeability measurement of polyester before and after coating with Prussian blue (PB) formulations at different concentrations (0.5 g/L, 1 g/L and 2 g/L), (n = 10, ±SD), **** *p* < 0.0001 (one-way ANOVA, Šídák’s test). The control used is referred to polyester samples without functionalisation.

As shown in Figure 2, regarding air permeability, the addition of the polymeric solution significantly altered the capacity of the air to pass through the knits. As exposed, when compared to the control without functionalisation, there is a decrease of approximately 800 L/m^2^/s for PB 0.5 g/L and 400 L/m^2^/s for PB 1 g/L and PB 2 g/L. These reductions of coated samples were to be expected given that a fabric’s air permeability is directly proportional to its air porosity [24] and fabric thickness [10], i.e., more porosity equals more permeable fabric, and the inverse relation is seen for thickness. Thus, after being coated, the fabrics’ pores are filled and the samples become thicker, having an impact on air permeability and leading to its reduction.

Higher values of air permeability are related to better breathability and heat dissipation, highly contributing to the thermal physiological comfort of the wearer. The use of low-permeability clothing causes inhibition of sweat evaporation and dissipation of thermal energy, leading to accumulation on the inner surface and subsequently discomfort [25]. For better comparison, the results obtained for this parameter were recalculated to different units (Table 2). Havenith et al. (2011) measured the air permeability of an NBC (nuclear, biological, chemical) suit generally used as protective equipment, reaching a value of 1115 L/min/cm^2^/bar [26], comparable to the one obtained for PES coated with PB 0.5 g/L. The values obtained for the samples PB 1 g/L and PB 2 g/L were almost twice that of the NBC suit, being able to provide a better comfort perception.

**Figure 3:**
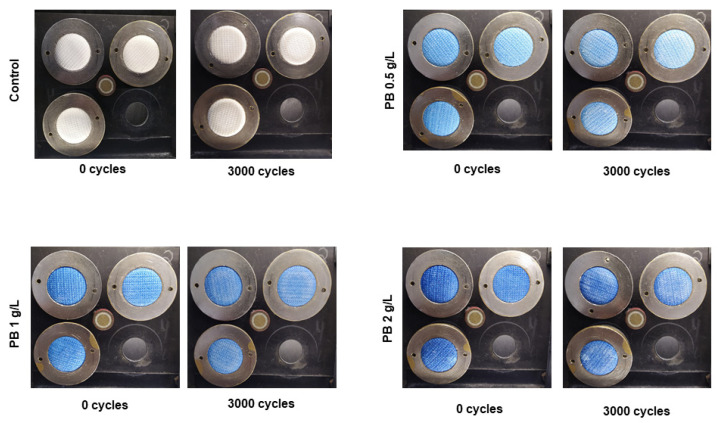
Visual results for polyester knits coated with Prussian blue (PB) at different concentrations (0.5 g/L, 1 g/L and 2 g/L) before and after 3000 cycles in the Martindale machine.

PB-coated samples were also evaluated for their abrasion resistance. Abrasion resistance, often called wear resistance, can be defined as the capacity of a material to resist rubbing or friction during its life cycle [27]. Apart from the effects on aesthetics, a material should present high abrasion resistance to prevent the loss of its initial functionality and maintenance of structural integrity [28]. In Figure 3, it is possible to see an image representation of functionalised polyester samples before and after 3000 abrasion cycles. For all concentrations, the textiles did not show any pilling signals, maintaining their structural integrity during the cycles. Additionally, weight loss percentages (Table 3) are equal to 0% for the three conditions tested and the control, indicating a high abrasion resistance. 

### 3.3. Preliminary Assessment of Prussian Blue-Coated Textiles Response to a Reducing Agent

**Figure 4:**
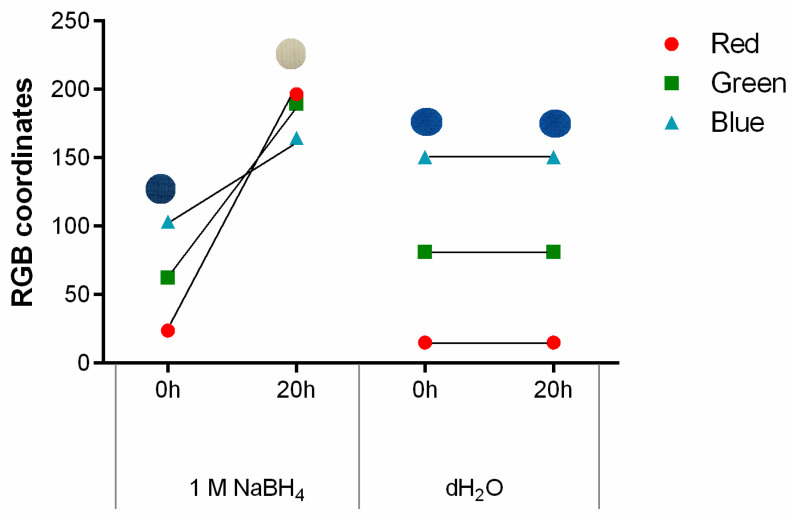
The g/L, after exposure to 1 M NaBH4, as a reducing agent, and distilled water, as a control, for 20 h, (n = 3, ±SD).

As previously mentioned, PB can detect living bacteria due to a reduction mechanism, causing the formation of its reduced form, Prussian white (PW), and the colour shift from blue to white [29]. Considering the functional properties of PB, the colour change induced by a reducing agent was tested after stamping on polyester with 1 g/L. Sodium borohydride (NaBH_4_) was used due to its strong reducing capacity [30]. As seen in Figure 4 and as expected, the contact with NaBH_4_ triggered the formation of PW and consequent modification in the colour displayed by the textile, confirmed by RGB. As the sample becomes whiter there is an increase in the value of all three coordinates, which was anticipated since the colour white is obtained when red, green and blue coordinates equal to 255. Furthermore, when it comes to the different channels, the red one has the highest slope/value variation, being the most sensitive to the colour change observed. This conclusion is in agreement with previous research work [22]. In contrast, the control used and represented as distilled water (dH_2_O) showed no signs of visible colour alteration, which was further confirmed by the RGB coordinates.

### 3.4. Evaluation of the Bacterial-Sensing Activity of Polyester Textiles Functionalised with Prussian Blue in Liquid and Solid Conditions

The bacterial-sensing activity was evaluated in liquid and solid mediums. For both assays, Gram-positive and Gram-negative bacteria were used, namely *S. aureus* and *E. coli*, respectively. 

**Figure 5:**
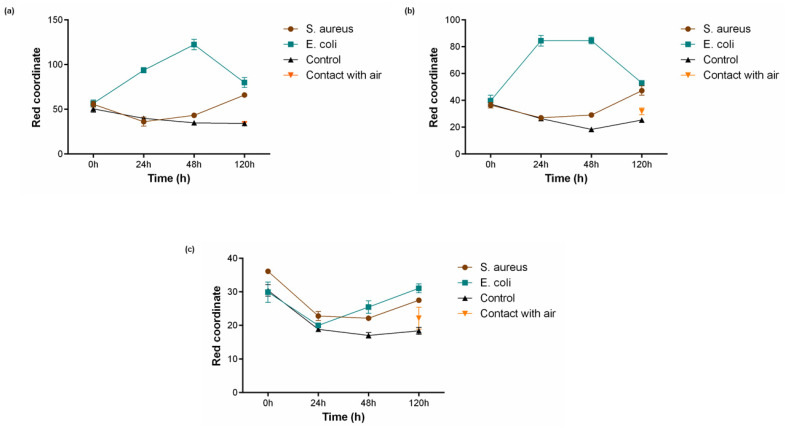
Bacterial sensing activity of polyester functionalised with Prussian blue (PB) at (**a**) 0.5 g/L, (**b**) 1 g/L and (**c**) 2 g/L. Colour change is represented by a shift in the red channel coordinate measured through the RGB model, after incubation with *S. aureus* (10^8^ CFU/mL) and *E. coli* (10^8^ CFU/mL) for 120 h in 1 M MES buffer (pH 6.1) and 1% (*w*/*v*) of glucose, (n = 3, ± SD). Control is represented as the condition where the textiles were incubated without bacterial culture and contact with air refers to a sample left in atmospheric conditions for the whole duration of the assay.

For the assessment of PB functionality in liquid conditions, textiles coated with different PB concentrations (0.5 g/L, 1 g/L and 2 g/L) were incubated in MES buffer (pH 6.1) supplemented with 1% (*w*/*v*) glucose. Additionally, two controls were performed, one without the presence of bacteria and another in contact with air. As shown in Figure 4, the two lowest concentrations 0.5 g/L (Figure 5a) and 1 g/L (Figure 5b) were capable to detect the presence of bacteria, both *S. aureus* after 120 h and *E. coli* after 24 h, which is reflected by an increase in value of the red coordinate measured. This activity is not influenced by the buffer used or the atmospheric air since the controls do not exhibit growing values for the red channel, having even a contrary tendency to decrease. After 48 h of incubation with *E. coli*, both concentrations show a drop in the red coordinate values measured, indicating a break in the tendency observed until that point. The Prussian blue–Prussian white redox mechanism is responsible for textile colour changes and bacterial detection. This is a reversible mechanism, meaning the compound can switch between the two forms in response to an external stimulus [31]. After 48 h, the supplemented glucose was already metabolised by the microorganism and, subsequently, due to lack of energy source, the bacteria start to die and Prussian white can be once again oxidised by the oxygen present in the atmosphere, forming Prussian blue [32]. This behaviour has also been reported in another study using PB as a sensor for bacteria detection [22]. 

On the other hand, polyester textiles coated with the highest concentration of 2 g/L (Figure 5c) did not display the capacity to respond to the presence of the microorganisms tested. For this condition, the red coordinate showed a primary decline and only started to increase after 48 h of incubation, not exceeding initial values. This lack of activity may be due to a concentration saturation, i.e., for concentrations higher than 1 g/L the bacteria are unable to reduce the necessary quantity of Prussian blue to produce a perceptible colour change.

When it comes to time response, the samples functionalised with 0.5 g/L and 1 g/L of PB presented a similar result, increasing in about 40 points for *E. coli* after 24 h of incubation and 10 points for *S. aureus* after 120 h. However, for the Gram-negative bacteria, it was noticed that the red coordinate for polyester textiles coated with PB 0.5 g/L continued to upsurge until 48 h. The distinct capability of the textiles to detect Gram-positive and Gram-negative bacteria is easily noticeable in Figure 4, with the last one generating a much quicker alteration in textile colouring. This can be justified by the morphological differences between the two microorganisms. Gram-negative bacteria possess several redox-active proteins in their outer membrane, and even if Gram-positive bacteria seem to have a simpler morphology, they can be characterised by the presence of a thick peptidoglycan non-conductive layer and a weak extracellular electron shuttling activity [33]. Thus, *S. aureus* exhibits slower extracellular electron transfer, leading to a slower reduction of PB. 

**Figure 6:**
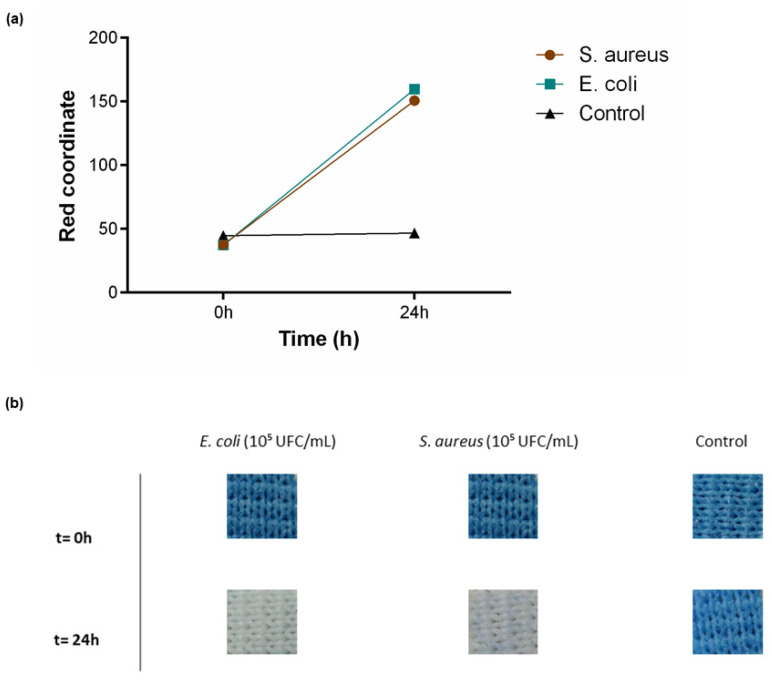
Bacterial sensing activity of polyester functionalised with Prussian blue (PB) at 1 g/L. (**a**) Shift in the red channel coordinate, measured through the RGB model, after incubation with *S. aureus* (10^5^ CFU/mL) and *E. coli* (10^5^ CFU/mL) for 24 h in TSA, (n = 3, ± SD). (**b**) Colour change displayed by the samples throughout the assay. Control is represented as the condition where the textiles were incubated only with TSA.

To test textile functionality in solid medium conditions, PB-coated polyesters were incubated in contact with TSA medium for 24 h. As previously described, *E. coli* was used as a Gram-negative bacteria and *S. aureus* as Gram-positive. A control with only TSA was also performed. Since above it was verified that both 0.5 g/L and 1 g/L could detect the presence of living bacteria, this assay was performed using the highest functional concentration and the sensitivity can be extrapolated to lower concentrations. As represented in Figure 6a, an increase in the red coordinate for both bacteria was observed, along with a visual modification of the textile from blue to white (Figure 6b). The control was as expected with no shift in the red channel or colour alteration throughout the analyses. 

It was proven that polyester knits functionalised with PB can sense both Gram-positive and Gram-negative bacteria in liquid and solid conditions. Thus, the studied textiles showed potential to be applied as patches in personal protective clothing in diverse areas, as needed. PB is a compound with great potential, with low cost, good biocompatibility characteristics and non-toxicity, being also studied in biomedical fields [34,35]. PB’s biocompatibility is mainly due to the bond between its cyano groups and iron ions, preventing any negative impact on cells [19,36]. Along with that, in contrast with other research works where PB is synthesised as nanoparticles and used as a sensor for diverse applications [29,37], our study demonstrated the functionality of commercial PB used as is, facilitating the acquisition of the functional molecule without the need for a complex synthesis.

### 3.5. Evaluation of Water Repellence and Chemical Penetration Resistance and Repellence

As the main goal of the present study was to develop a patch to be placed on protective equipment, along with the contact angle assessment, the capacity of the textile to resist chemical penetration and display repellence to both acidic and alkaline solutions was assessed.

**Figure 7:**
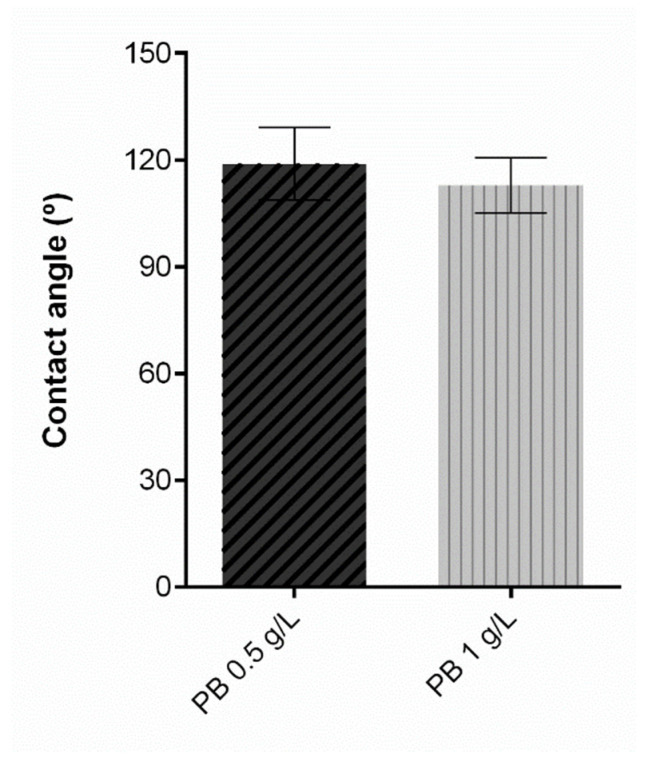
Contact angle measurement of polyester after coating with Prussian blue (PB) formulations at different concentrations (0.5 g/L, 1 g/L and 2 g/L), (n = 10, ± SD).

Regarding the contact angle, protective textiles must at least meet the hydrophobicity threshold to prevent the passage of contaminated liquids and consequentially contact with the skin. Contact angle measurements reflect a solid’s ability to repel a liquid, and thus higher contact angles are associated with a superior repellent effect. [38]. A textile surface is hydrophobic if the water contact angle is higher than 90°, and its hydrophobicity increases in proportion with its contact angle [39], becoming superhydrophobic if it is more than 150° [40]. Contact angle results for the samples under study are exposed in Figure 7. The control knit, consisting of untreated polyester is not presented in the graphic since the water drop was instantly absorbed and the contact angle could not be measured, from which we can deduce a hydrophilic behaviour. On the contrary, after the functionalisation process with PB, all knits presented a hydrophobic performance, proving that coating with a polymeric formulation allows for obtaining satisfactory results for this property. As previously stated, the polymeric base is primarily polyurethane, and after coating, the substrates are subjected to a temperature-controlled drying step, which allows the formation of a film on the knit surface and hence its hydrophobic behaviour. The functionalised samples do not present significant differences between them, which indicates that PB concentration does not affect this parameter.

To evaluate chemical protection, an assay based on ISO 6530 was carried out to measure the penetration index and repellence of the materials against liquid chemicals, especially those of low volatility. As chemical agents, an acid, H_2_SO_4_ 30% (*v*/*v*), and a base, NaOH 10% (*w*/*v*), were used. In this test, the samples with a concentration of 0.5 g/L and 1 g/L were analysed, since they were the ones that showed bacterial-sensing potential. The level of performance is subsequently evaluated according to the above-mentioned criteria in Table 1. For the acid agent, the results seen in Table 4 show a low penetration index and a high repellence efficiency, with the sample PB 0.5 g/L displaying level 3 performance for both measurements. For PB 1 g/L the performance level for penetration is classified as 2, however, the index percentage is near the threshold value (1%). For the repellence index, the percentage obtained was above 100, which was not expected since the fibrous surface cannot repel more solution than what is poured on top of it. This could be explained by a small degradation/corrosion of the fibres, caused by the sulfuric acid at high concentration. Under optimal conditions of temperature, time and concentration this acid is frequently used in the hydrolysis of polyester structures [41,42], which indicates the potential of this chemical to degrade this material. 

Concerning textile behaviour in contact with an alkaline agent, the results presented in Table 5 show a level 3 penetration and repellence performance from both samples under evaluation. 

Overall, PB-coated samples demonstrated a high penetration resistance and repellence effect for both acid and base chemicals, making them suitable to use as a patch attached to PPE. These results can be further analysed in correlation with the air permeability measures demonstrated in Section 3.1, where it was observed higher values of breathability for the sample PB 1 g/L than for PB 0.5 g/L and the NBC suit referred. Hence, chemical penetration resistance and repellence evaluation validate that even with higher air permeability, the protection of the structure functionalised with PB 1 g/L has not been compromised.

## 4. Conclusions

Herein, Prussian blue (PB) was evaluated as a functional compound applied on polyester knits to act as an on-site sensor for bacteria detection. The bacterial sensing capacity of the coated knits was assessed in a liquid medium, with the two lowest concentrations tested (0.5 and 1 g/L) exhibiting the capability of detecting both Gram-positive and Gram-negative bacteria. Furthermore, PB 1 g/L was also able to act as a sensor in a solid medium, changing from blue to white. Thus, the responses given by the samples are clear and easily perceptible by the operator. Through this study, it is possible to verify the existence of a saturation point, after which PB ceases to demonstrate functionality. 

Evaluation of water repellence and chemical penetration resistance and repellence was also performed in polyester functionalised with PB 0.5 and 1 g/L. Both knits showed a hydrophobic behaviour and a capacity to resist to penetration of chemicals and level 3 repellence effect for both acid and base chemicals, demonstrating the potential application in PPE. The functionalised samples displayed a decrease in air permeability values, in comparison to the control PES, as well as high abrasion resistance (>2000 cycles).

For future research, it would be of great interest to determine the minimum bacterial concentration required to trigger a response by the sensor, as well as evaluate and optimise the response time of the developed sensor after encountering bacteria. Furthermore, due to the intended end use, repellence and penetration resistance to fake blood should be studied. It is also important to assess other conditions affecting the durability of the sensors on PES, i.e., wash resistance, light fastness and sensing activity after storing the samples for a set period. If needed, durability improvements could be made through chemical modification of the PES substrate, for example using mordants and leading to a better fixation of the functional PB. 

## Figures and Tables

**Table 1:** Repellence and penetration indexes, in percentage, and respective performance level, according to ISO 14325.

	Performance Level		
	3	2	1
Repellence Index (%)	>95	>90	>80
Penetration Index (%)	<1	<5	<10

**Table 2:** Air permeability values of the control polyester before coating and the samples functionalised with Prussian blue (PB) formulations at different concentrations (0.5 g/L, 1 g/L and 2 g/L) converted into different units.

Air Permeability Units	Control	PB 0.5 g/L	PB 1 g/L	PB 2 g/L
L/m^2^/s (@200 Pa)	1156	288.8	733.2	713.2
L/min (@200 Pa)	138.7	34.7	88.0	85.6
L/min/cm^2^/bar	3467.5	867.5	2200.0	2140.0

**Table 3:** Weight loss percentage of polyester knits coated with Prussian blue (PB) at different concentrations (0.5 g/L, 1 g/L and 2 g/L) after 3000 cycles in the Martindale machine, (n = 3, ± SD).

	Weight Loss ± SD (%)
Control	0.00 ± 0.00%
PB 0.5 g/L	0.00 ± 0.00%
PB 1 g/L	0.00 ± 0.00%
PB 2 g/L	0.00 ± 0.00%

**Table 4:** Values of penetration index (%) and repellency index (%) for polyester knits functionalised with Prussian blue (PB) at 0.5 and 1 g/L, after contact with H_2_SO_4_ 30% (*v*/*v*), (n = 3, ±SD). Performance levels were attributed to penetration and repellence indexes according to ISO 14325.

H_2_SO_4_ 30% (*v*/*v*)				
	Penetration Index ± SD (%)	Penetration Performance Level	Repellence Index ± SD (%)	Repellence Performance Level
PB 0.5 g/L	0.0 ± 0.0	3	104.6 ± 5.5	3
PB 1 g/L	1.3 ± 1.1	2	103.2 ± 3.6	3

**Table 5:** Values of penetration index (%) and repellency index (%) for polyester knits functionalised with Prussian blue (PB) at 0.5 and 1 g/L, after contact with NaOH 10% (*w*/*v*), (n = 3, ±SD). Performance levels were attributed to penetration and repellence indexes according to ISO 14325.

NaOH 10% (*w*/*v*)				
	Penetration Index ± SD (%)	Penetration Performance Level	Repellence Index ± SD (%)	Repellence Performance Level
PB 0.5 g/L	0.0 ± 0.0	3	100.8 ± 4.3	3
PB 1 g/L	0.7 ± 0.6	3	96.6 ± 1.3	3

## Data Availability

Not applicable.
